# DOES CHATGPT HAVE AGEISM?

**DOI:** 10.1093/geroni/igae098.3270

**Published:** 2024-12-31

**Authors:** Huai Cheng

**Affiliations:** Minneapolis VA Hospital, Playmouth, Minnesota, United States

## Abstract

Ageism is prevalent in the media and clinical practice. AI is defined as thinking and acting like humans. ChatGPT as one type of AI was released to public in 11/2022 and immediately emerged as a quick information resource and interaction with humans. It is very important to know whether ChatGPT has discrimination against older adults and offers non-age biased health information in aging and geriatrics. To explore these, ChatGPT’s responses to geriatric attitudes inquiry were assessed by using a validated instrument. This geriatrics attitude instrument is composed of 6 positive attitude questions and 10 items negative attitude questions. They have been tested among medical students, residents, and geriatric fellows. In the present study, ChatGPT was prompted to answer these 16 geriatric attitude questions on a scale of 1-5 (1-strongly disagree and 5=strongly agree). Prompt is based on the directions and questions from the originally published paper. The outputs from ChatGPT were collected to compare the results from medical students, residents and geriatric fellows published in the literature. The total geriatrics attitude score from ChatGPT was significantly lower than humans (2.68 vs 3.77). However, the mean positive geriatric attitude score was 4.1 (the higher the better). The mean negative geriatric score was 1.8 (the lower the better). About half outputs from ChatGPT also provide positive rational for their responses. Our results demonstrate that ChatGPT is less likely to produce an age-biased output. Therefore, we should be comfortable to trust non-age biased output on aging and geriatrics from ChatGPT.

